# Advanced glycation end-product intake predicts insulin resistance in a sex-dependent fashion

**DOI:** 10.1007/s00394-025-03672-3

**Published:** 2025-04-22

**Authors:** Domenico Sergi, Sharon Angelini, Riccardo Spaggiari, Fabiola Castaldo, Giovanni Zuliani, Juana Maria Sanz, Angelina Passaro, the PANGEA study group, Edoardo Dalla Nora, Gloria Brombo, Eleonora Capati, Cecilia Soavi, Rosella Colonna, Elettra Mantovani, Mario Luca Morieri, Maria Agata Miselli, Alice Omenetto, Sefora Del Mastro, Gabriella Stifani, Daniela Francesconi, Stefano Lazzer, Giovanelli Nicola, Mirco Floreani, Martina Arteni, Alberto Botter, Desy Salvadego, Gianni Biolo, Roberta Situlin, Filippo Giorgio Di Girolamo, Mariella Sturma, Giuseppe Castiglia, Marcello Tence, Greta Del Fabbro, Sara Mazzucco, Paolo De Colle, Boštjan Šimunič, Rado Pišot, Uroš Marušič, Matej Plevnik, Saša Pišot, Dorjana Zerbo, Nina Mohorko, Petra Dolenc, Mojca Gabrijelčič Blenkuš

**Affiliations:** 1 Department of Translational Medicine, University of Ferrara, 44121 Ferrara, Italy; 2 Department of Chemical, Pharmaceutical and Agricultural Sciences, University of Ferrara, 44121 Ferrara, Italy

**Keywords:** Advanced glycation end products, Insulin resistance, Sex-dependent effect of diet

## Abstract

**Purpose:**

Dietary advanced glycation end products (AGEs) have been implicated in promoting insulin resistance. However, their impact on insulin resistance in a mixed population made up of males and females remains controversial. The aim of this study was to evaluate whether the relationship between dietary AGEs and insulin resistance may be sex-dependent.

**Methods:**

195 males and 239 females were included in this cross-sectional study. Study participants underwent anthropometric and metabolic assessments. AGE intake was estimated using food frequency questionnaires and databases reporting AGE content in individual food items. The relationship between AGE intake and insulin resistance, estimated using HOMA-IR, was assessed using Pearson correlation test. The predictive power of dietary AGEs towards HOMA-IR was investigated using stepwise linear regression.

**Results:**

AGE intake correlated positively with HOMA-IR in females (p < 0.01) but not in male study participants (p > 0.05). Moreover, AGE intake was able to increase the predictive power of BMI towards insulin resistance in females but not males. Instead, anthropometric variables were the only discriminants able to predict insulin resistance in males.

**Conclusion:**

Dietary AGEs exert a sex-dependent effect on insulin resistance as their intake is associated with and able to predict HOMA-IR in females but not males. This suggests that females may be more susceptible to the deleterious impact of these glycotoxins on insulin sensitivity. Nevertheless, considering this study not involving a nutritional intervention to directly elucidate whether the effect of AGEs on insulin resistance is sex-dependent, further studies are warranted to confirm the present findings.

**Supplementary Information:**

The online version contains supplementary material available at 10.1007/s00394-025-03672-3.

## Introduction

Developed and increasingly developing countries are facing a diabetes epidemic predominantly driven by the exponential increase in the cases of type 2 diabetes mellitus (T2DM) [1]. Obesity, particularly central adiposity, is a key risk factor for T2DM in light of its close relationship with insulin resistance, the hallmark of T2DM [2]. From a pathogenetic perspective, obesity contributes to insulin resistance by fostering metabolic inflammation and lipotoxicity which, in turn, are two pivotal mechanisms in impairing insulin signaling [3–5]. In this context, the Western diet is crucial, not only because it is the main modifiable and independent risk factor [6], but also because of its role in supporting the chronic low-grade inflammation typical of obesity [7] and in promoting lipotoxicity [8]. The consumption of highly processed foods represents one of the key actors in mediating the metabolic aberrations associated with the Western diet. Indeed, they have been implicated in promoting body weight gain and obesity [9], T2DM [10] and increased cardiovascular risk [11]. Although highly processed foods are high in long-chain saturated fatty acids and sugars and low in dietary fiber, these nutrients may not be the only mediators of their effect on metabolic health and insulin resistance in particular. Indeed, food processing also affects the nutritional quality of foods by promoting the formation of advanced glycation end-products (AGEs) which, in turn, are another link between the Western diet and poor metabolic health [12].

AGEs represent a heterogeneous group of molecules produced non-enzymatically as a consequence of the interaction between reducing sugars and the free amino groups of proteins, nucleic acids, and lipids via the Maillard reaction during dry heat food processing, including cooking [13]. Besides heat processing, AGE formation is dependent on the nutritional composition of food items, with AGE content being higher in foods rich in fat and proteins [13]. Considering their heterogeneity, AGEs include molecules like carboxyethyl lysine, carboxymethyl lysine, glyoxal lysine dimer, 3-deoxyglucosone lysine dimer and pyrroline [12]. AGEs introduced with foods, particularly low-molecular weight AGEs, are absorbed in the small intestine [14] and their biological effects are mediated by the activation of the receptor for AGEs (membrane RAGE, mRAGE; soluble RAGE, sRAGE) [15] which culminate with the induction of oxidative stress and the pro-inflammatory pathways, namely c-Jun N-terminal kinase and the factor nuclear-factor-kappa B (NF-κB) [16–18].

From a metabolic perspective, AGEs have been reported to promote insulin resistance in vivo in rats [16] and mice [19] as well as in vitro in rodent skeletal muscle cells [16] and 3T3-L1 adipocytes [19]. Remarkably, a diet low in AGEs increased insulin sensitivity in overweight but otherwise healthy individuals [20] and in individuals with T2DM [21], further supporting the implications of dietary AGEs on insulin resistance. In humans and animal models, AGEs intake effects on insulin resistance remain controversial; indeed, other studies have not identified a relationship between habitual AGEs intake and insulin sensitivity [22, 23]. The reason for these discrepancies may be related to the fact that females appear to be more susceptible to the metabolically detrimental effects of AGEs. In support of this, while studies only including males did not identify a relationship between serum AGEs and insulin sensitivity [24], in study cohorts only made up of overweight women, AGE restriction improved insulin sensitivity [25]. Additionally, the knock of the RAGE resulted in an improvement in insulin sensitivity in female but not in male mice fed a high-fat diet [26].

Thus, despite dietary AGEs being implicated in promoting insulin resistance in mixed male and female study cohorts, their effects are controversial and may be sex-specific. In light of this, the aim of this study was to evaluate the relationship between AGE intake and insulin resistance and if the ability of these glycotoxins to predict deterioration of insulin sensitivity is sex-dependent.

## Subjects and methods

### Participants

Participants in the cross-sectional PANGeA (Physical Activity and Nutrition for Quality Aging) project were considered for this study [27]. The study conducted in northern Italy, recruited free-living individuals aged between 55 and 80 years and able to walk for 2 km independently. Subjects with anticoagulant therapy or previous cancer diagnosis or hospitalization in the last year or missing were excluded. After the exclusion of twenty-five PANGeA study participants without food frequency questionnaire data, four hundred and thirty-four participants (195 males and 239 females) were included in this study.

Subjects enrolled gave their written consent to participate in the study and were subjected to anamnestic and nutritional interviews, anthropometric measurements and a blood sampling as previously described [27] and detailed below.

### Dietary assessment and semi-quantitative dietary AGEs evaluation

Study participants completed a 90-item food frequency questionnaire with indications of food portion sizes. Dietary AGE intake was estimated for each participant using their food intake frequencies, after adjusting portion sizes for study participant total energy intake, and the AGE content of food samples reported in previously published databases [13, 28]. For some food items, it was not reported the cooking method in the food frequency questionnaire. In this case, the AGE content of the food was expressed as the mean of the most common cooking methods used for that specific item. Then, the AGE intake for each participant was obtained by multiplying the frequency of single food item, referred to as frequency of consumption over a year, by the mean AGE content, expressed as kiloUnits (kU) per serving of each food item consumed by study participants. Finally, the AGEs intake for each participant was expressed as kU AGEs/day.

Supplements if consumed were not considered for the estimation of AGEs intake. Nutrient and energy intake were assessed using 24 h recalls. The adherence to the Mediterranean Diet was computed using a Mediterranean Diet adherence score (MDA) as detailed previously [27].

### Biochemical analysis

Blood samples were collected after an overnight fast and centrifuged at 1600 g for 15 min at 4 °C to obtain serum or EDTA plasma. Aliquots were stored at −80 °C until use. Serum triglycerides, total cholesterol, HDL cholesterol (HDL), glucose and insulin concentrations were measured by standard enzymatic-colorimetric methods. LDL cholesterol (LDL) levels were computed by the Friedewald's formula [29]:

$$LDL\,cholesterol = Total\,cholesterol - HDL\,cholesterol - \frac{{Tryglicerides}}{5}$$

Serum High-sensitivity C-reactive protein (hsCRP) was measured by immune-turbidimetry (CRP OSR6147, Beckman Coulter, Brea, CA, USA). Insulin resistance was calculated using the Homeostasis model assessment index (HOMA-IR) formula [30]:

$$HOMA - IR\,index = \frac{{Glucose\,(mg/dL)*Insulin\,(mU/L)}}{{405}}$$

Visceral adipose Index (VAI) was estimated according to Amato et al. [31] as follows:

For female

$$VAI = \frac{{Waist\,circumference\,(cm)}}{{((1.89*BMI) + 36.58)}}*\frac{{Tryglicerides\,(mg/dl)}}{{0.81}}*\frac{{1.52}}{{HDL\,cholesterol\,(mg/dl)}}$$

For male

$$VAI = \frac{{Waist\,circumference\,(cm)}}{{((1.88*BMI) + 39.68)}}*\frac{{Tryglicerides\,(mg/dl)}}{{1.03}}*\frac{{1.31}}{{HDL\,cholesterol\,(mg/dl)}}$$

(BMI = body mass index).

### Anthropometric measurements and body composition

Anthropometric measures were performed on participants wearing light clothing and no restrictive underwear nor shoes. Body weight was rounded to the nearest 100 g whereas height and waist circumferences were all rounded to the nearest 0.1 cm. Waist circumference was measured between the lowest rib and iliac crest around the smallest circumference.

Bioelectrical impedance (tetrapolar impedance meter, BIA101, Akern, Florence, Italy) was used to determine body composition: fat mass (FM), fat-free mass (FFM) and muscle mass. Bioimpedance was performed by a trained staff member on subjects in a horizontal position, after 8 h of fasting.

### Statistical analysis

Continuous variables were analyzed using Shapiro–Wilk tests. Non-normally distributed variables were expressed as median (Quartile 1–Quartile 3). The comparison of variables between males and females was carried out with Mann–Whitney tests for non-normally distributed parameters. Pearson correlation analysis was used to evaluate the association between AGEs or HOMA-IR and the anthropometric and metabolic parameters. Stepwise multiple regression analysis was performed to assess the predictive power of AGEs and other parameters of interest for HOMA-IR. In these analyses, the variables not normally distributed were log-transformed. Data analysis was performed using SPSS Statistics for Windows, version 26.0 (SPSS, Inc., Chicago, IL) and a p ≤ 0.05 was considered statistically significant.

The missing data for each variable of interest did not exceed 5%.

## Results

### General, anthropometric, metabolic characteristics and AGE intake of the study participants

The characteristics of the study participants divided by sex are reported in Table 1. As expected, male, compared to female study participants, had a higher BMI, waist circumference, FFM, and systolic and diastolic blood pressure (all p ≤ 0.001) **(**Table 1). On the contrary, FM in percentage but not in Kg, and VAI (p = 0.007) was higher in females compared to males (p < 0.001) **(**Table 1**)**. From a metabolic perspective, total, LDL and HDL cholesterol was higher in females than in males (all p < 0.001), whereas fasting blood glucose (p < 0.001), blood insulin (p = 0.012) and HOMA-IR (p < 0.001) were higher in males (Table 1). Finally, the consumption of AGEs adjusted for energy intake was higher in males relative to females (p = 0.003) (Table 1). Furthermore, the number of individuals with hypertension, taking anti-hypertensive drugs or metformin was higher among males compared to females (Table 1).

**Table 1:** General characteristics of male and female study participants

	Male (n = 195)	Female (n = 239)	p-value^1^
	Median (IQR)	Median (IQR)	
Age (years)	66 (63–70)	65 (63–70)	0.103
BMI (kg/m^2^)	26.8 (24.7–29.3)	25.7 (23.2–28.2)	0.001***
Waist circumference (cm)	96.0 (90.0–103.0)	89.0 (83.0–96.0)	< 0.001***
FFM (%)	68.8 (65.1–72.5)	61.2 (57.1–64.8)	< 0.001***
FM (%)	31.2 (27.5–34.9)	38.8 (35.2–42.9)	< 0.001***
FFM (Kg)	54.7 (51.5–58.6)	39.2 (36.6–42.3)	< 0.001***
FM (Kg)	24.5 (20.7–29.7)	24.82 (20.6–29.5)	0.907
Muscle Mass (Kg)	34.9 (32.0–37.3)	23.9 (21.9–26.0)	< 0.001***
VAI	0.91 (0.61–1.46)	1.0 (0.81–1.51)	0.007**
SBP (mmHg)	143 (132–158)	133 (120–146)	< 0.001***
DBP (mmHg)	88 (81–94)	82 (76.67–90.)	< 0.001***
AGEs (kU/day)	11,925.2 (8811.6–17,030.5)	10,492.7 (8103.9–14,692.8)	0.003**
Glucose (mg/dL)	100 (91–110)	94 (88–102)	< 0.001***
Insulin (U/L)	8.8 (6.1–12.4)	7.50 (5.6–10.0)	0.012*
HOMA-IR index	2.2 (1.4–3.4)	1.7 (1.2–2.4)	< 0.001***
Triglycerides (mg/dL)	94 (70–125)	91 (71–116)	0.747
Total Cholesterol (mg/dL)	200 (179–228)	226 (204–250)	< 0.001***
Cholesterol HDL (mg/dl)	57 (48–69)	72 (61–81)	< 0.001***
Cholesterol LDL (mg/dl)	122 (100–143)	136 (113–156)	< 0.001***
hsCRP (mg/L)	0.11 (0.06–0.21)	0.10 (0.06–0.26)	0.792
IL-18 (pg/ml)	400.9 (319.6—503.5)	320.9 (263.0—405.8)	< 0.001***

	N (%)	N (%)	p-value^2^
Hypertension	72 (36.9%)	63 (26.4%)	0.022*
Hypertension therapy	69 (35.4%)	62 (26.4%)	0.036*
Hypolipidemic treatment	43 (22.1%)	35 (14.6%)	0.059
Metformin therapy	13 (6.7%)	3 (1.3%)	0.004**
Smoke	16 (8.3%)	25 (10.6%)	0.267

The comparison between male and female was carried out with Mann–Whitney test^1^ or Fisher Exact Test^2^

*0.050 > p-value ≤ 0.010; **0.010 > p-value < 0.001; ***p-value ≤ 0.001

BMI, body mass index; FFM, fat free mass; FM, fat mass; VAI, visceral adiposity index; SBP, systolic blood pressure; DBP, diastolic blood pressure; AGEs, advanced glycation end products; HOMA-IR, homeostatic model assessment for insulin resistance; HDL, high density lipoprotein; LDL, low density lipoprotein; hsCRP, high-sensitivity C-reactive protein; IL-18, interleukin-18

In terms of nutrient and energy intake, males consumed more energy, carbohydrates, proteins and lipids than females (Table S2). However, diet quality expressed as MDA did not differ between the two groups (Table S2).

### The relationship between AGE intake, anthropometric and metabolic health-related parameters

**Fig. 1:**
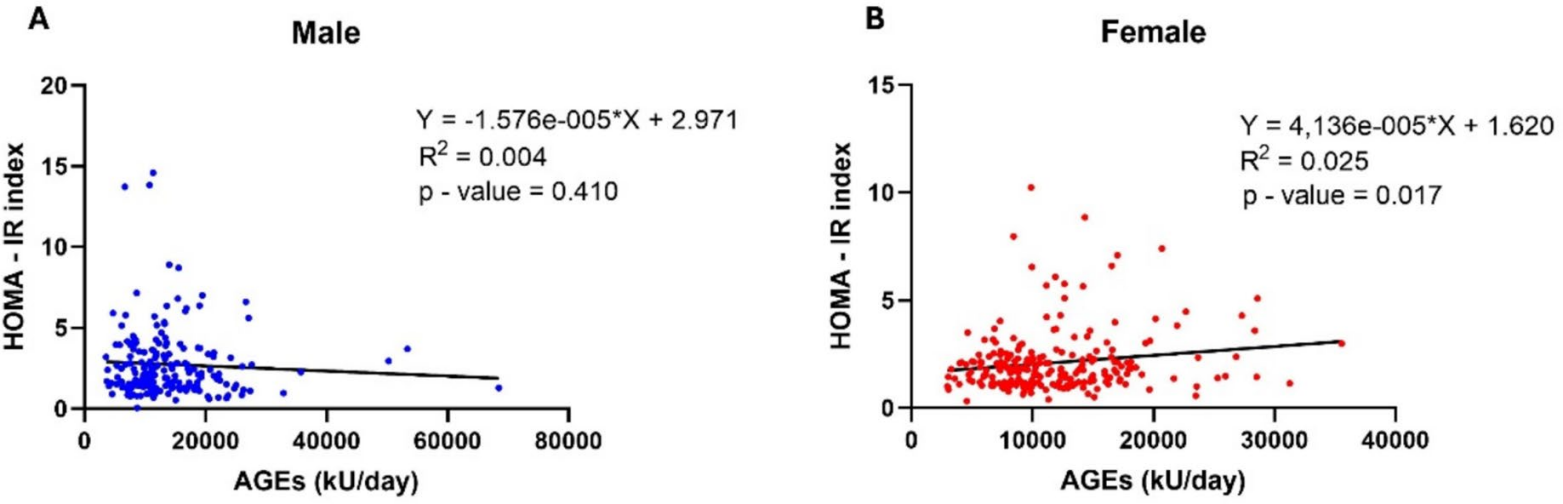
Correlation between daily AGE intake and insulin resistance in both sexes. On one side, the dot plot chart shows the absence of correlation between daily AGE intake and HOMA-IR index in the male population (**A**), while, on the other side, it reports a positive correlation between the same parameters in the female population (**B**). HOMA-IR homeostatic model assessment of insulin resistance, AGEs advanced glycation end product

Considering the relationship between dietary AGEs, insulin resistance and obesity [32, 33] which, however, remains controversial [22], it was investigated whether the habitual AGE consumption correlated with anthropometric and metabolic health-related parameters. The intake of AGEs in the whole study cohort correlated positively with FFM and negatively with visceral adiposity index (VAI), fasting triglycerides, total and LDL-cholesterol. Additionally, AGE intake did not correlate with fasting glycemia nor HOMA-IR (Table S3). However, considering that the present study cohort was made up of both males and females, to evaluate whether AGE intake affected differently males and females, the relationship between AGE intake and metabolic parameters was investigated in both sexes separately. While dietary AGEs in male correlated with fasting triglycerides (p = 0.034) but not with insulin resistance (Table 2; Fig. 1A), in females the intake of AGEs correlated positively with fasting blood insulin (p = 0.034) and HOMA-IR (p = 0.022) **(**Fig. 1B**)** and negatively with total (p = 0.005) and LDL-cholesterol (p = 0.002) **(**Table 2**)**. Additionally, in females dietary AGEs tended to correlate positively with fasting blood glucose (p = 0.065).

**Table 2:** Pearson’s correlation between AGEs, anthropometric and metabolic parameters of interest

	log AGEs (kU/day)			
	Male (n = 195)		Female (n = 239)	
	r	p-value	r	p-value
log BMI (kg/m^2^)	−0.062	0.390	−0.050	0.445
log Waist circumference (cm)	−0.020	0.781	−0.045	0.485
log FM (Kg)	−0.133	0.065	−0.023	0.724
log FFM (Kg)	−0.002	0.976	0.034	0.597
log VAI	−0.099	0.173	−0.070	0.299
log Triglycerides (mg/dL)	−0.153	0.034*	−0.064	0.339
log Total Cholesterol (mg/dL)	−0.013	0.860	−0.185	0.005**
log Cholesterol HDL (mg/dl)	0.002	0.973	0.056	0.403
log Cholesterol LDL (mg/dl)	0.036	0.616	−0.207	0.002**
log Glucose (mg/dL)	−0.082	0.261	0.123	0.065
log Insulin (U/L)	−0.026	0.726	0.142	0.034*
log HOMA-IR index	−0.047	0.518	0.153	0.022*
log IL-18 (pg/ml)	0.081	0.261	−0.043	0.521
log hsCRP (mg/L)	−0.006	0.931	−0.057	0.394

*0.050 > p-value ≤ 0.010; **0.010 > p-value < 0.001

*r,* Pearson Correlation Coefficient; BMI, body mass index; FFM, fat free mass; FM, fat mass; VAI, visceral adiposity index; SBP, systolic blood pressure; DBP, diastolic blood pressure; AGEs, advanced glycation end products; HOMA-IR, homeostatic model assessment for insulin resistance; HDL, high density lipoprotein; LDL, low density lipoprotein; hsCRP, high-sensitivity C-reactive protein; IL-18, interleukin-18

### AGE intake as a predictor of HOMA-IR

Given the relationship between AGE intake and HOMA-IR in female study participants, it was next investigated whether dietary AGEs could predict insulin resistance and how their predictive power compared with known risk factors for insulin resistance [34]. It was first confirmed that in this study cohort existed a relationship between BMI, fat mass, VAI and HOMA-IR. As expected, all these variables correlated positively with HOMA-IR in the whole cohort (Table S4) as well as in males (Table 3) and females (Table 3) separately (all p < 0.001).

**Table 3:** Pearson’s correlation between HOMA-IR index and classical predictors for insulin resistance in male and female study participants

	log HOMA-IR index			
	Male (n = 195)		Female (n = 239)	
	r	p-value	r	p-value
log BMI (kg/m^2^)	0.518	< 0.001***	0.461	< 0.001***
log VAI	0.387	< 0.001***	0.417	< 0.001***
log FM (Kg)	0.564	< 0.001***	0.439	< 0.001***

^***^p–value ≤ 0.001

*r,* Pearson Correlation Coefficient; BMI, body mass index; FM, fat mass; VAI, visceral adiposity index

Considering BMI, FM and VAI all correlated positively with HOMA-IR when considering the whole study cohort, it was next assessed whether AGE intake could increase the predictive power of these variables towards insulin resistance. In the whole study cohort, the BMI was the primary predictor of HOMA-IR (p < 0.001), with its predictive power being increased by VAI (p < 0.001) in model 2, AGE intake (p < 0.005) in model 3 and FM (p = 0.007) in model 4 (Table S5). However, when considering only male study participants AGE intake failed to increase the predictive power of fat mass and VAI towards insulin resistance (Table 4A). On the contrary, in females, dietary AGEs were able to increase the capacity of BMI and VAI to predict variations in HOMA-IR (p = 0.001) (Table 4B).

**Table 4:** Stepwise linear regression model indicating predictors of HOMA-IR index in male (A) and female (B) study participants

(A) Male (n = 195)					
Model	Predictor	R2	Unstandardized B coefficient	Standard error	p-value
1	log FM (Kg)	0.318	−1.641	0.211	< 0.001***
			1.426	0.152	< 0.001***
2	log FM (Kg) log VAI	0.358	−1.373	0.220	< 0.001***
			1.238	0.157	< 0.001***
			0.229	0.067	0.001**
Dependent variable: log HOMA-IR					
Model 1 excluded variables: log AGEs (kU/day), log BMI (Kg/mq), log VAI Model 2 excluded variables: log AGEs (kU/day), log BMI (Kg/mq)					

(B) Female (n = 239)					
Model	Predictor	R2	Unstandardized B coefficient	Standard Error	p-value
1		0.213	−2.263	0.326	< 0.001***
	log BMI		1.780	0.230	< 0.001***
2		0.307	−1.825	0.317	< 0.001***
	log BMI		1.459	0.224	< 0.001***
	log VAI		0.327	0.060	< 0.001***
3		0.343	−2.735	0.407	< 0.001***
	log BMI		1.468	0.219	< 0.001***
	log VAI		0.340	0.058	< 0.001***
	log AGEs		0.223	0.065	0.001***
Dependent variable: log HOMA-IR					
Model 1 excluded variables: log AGEs (kU/day), log FM (Kg), log VAI Model 2 excluded variables: log AGEs (kU/day), log FM (Kg) Model 3 excluded variables: log FM (Kg)					

***p-value ≤ 0.001

BMI, body mass index; FM, fat mass; VAI, visceral adiposity index; AGEs, advanced glycation end products

## Discussion

The data reported herein describe a sex-dependent effect of dietary AGEs on insulin resistance. In particular, dietary AGEs are positively associated with and are able to predict HOMA-IR in female but not male study participants. Remarkably, the intake of AGEs was able to increase the predictive power of anthropometric variables known to negatively impact upon insulin sensitivity only in females.

The present data is in agreement with previous studies which reported that a diet low in dietary AGEs is able to improve insulin sensitivity in overweight women [25]. Other studies have also shown an inverse relationship between AGE intake and insulin sensitivity, albeit this relationship was described in mixed cohorts consisting of both males and females [20, 21], which does not allow to discriminate the contribution of each sex. At the same time, in other studies AGE intake did not affect insulin sensitivity [22, 23]. However, none of these studies evaluated whether these glycotoxins elicited a sex-dependent effect on insulin resistance. Thus, to our knowledge, this is the first study dissecting the role of sex on the relationship between AGE intake and insulin resistance. Indeed, despite in the first instance the present result confirming the ability of AGE intake to predict HOMA-IR, this effect disappeared when only males were included in the analysis. This suggests that the impact of habitual AGE intake on insulin resistance in this cohort is driven by females who, compared to males, appear more susceptible to the metabolic effects of dietary AGEs. This sex-dependent response to AGEs was also confirmed in another study, which despite not directly investigating insulin resistance, still reported that circulating AGEs were able to predict all-cause, cardiovascular disease, and coronary heart disease mortality in women but not in men [35]. Additionally, while in study cohorts only made of men serum AGEs were not linked with insulin resistance [24], in women dietary AGE restriction resulted in an improvement in insulin sensitivity [25]. Importantly, the intake of AGEs as part of this study is in line with previously published reports [36, 37] and above (≥ 10,000 kU/day) the value set for a low AGE diet in AGE-restricted dietary intervention studies [21, 38]. As such, the amount of AGEs consumed by both groups should be sufficient to identify a relationship with insulin resistance, further underlining that the effect of AGEs may be sex-specific.

However, the reason underlying the sex-dependent effect of AGEs on insulin resistance in the present and other studies remains to be fully elucidated, even though it may rely on the modulation of RAGE expression by estrogens. Indeed, 17-β-estradiol is able to upregulate mRAGE expression in human vascular endothelial cells [39]. At the same time, menopausal hormone replacement therapy with estradiol and norethisterone acetate led to a decrease in sRAGE [40]. Thus, estrogens, on one hand upregulate mRAGE leading to an increase in AGE-mediated intracellular signal transduction while, on the other hand, also increase AGE bioavailability by downregulating sRAGE [12, 41]. These effects of estrogens, in turn, would amplify the activation of the c-Jun N-terminal kinase and the transcription factor nuclear-factor-kappa B (NF-κB) pro-inflammatory pathways, the induction of oxidative stress and the downregulation of sirtuin1 [16–19, 42] which have all been implicated in the pathogenesis of insulin resistance [5, 43–45]. Nevertheless, the women included in this study were in the post-menopausal state and did not receive any hormone-replacing therapy which may have prevented an oestrogen-dependent modulation of both mRAGE and sRAGE. Despite this, the sex-dependent response to dietary AGEs, described herein, may still be driven by residual endogenous estrogenic milieu, as demonstrated by its effect on bone mass in postmenopausal women [46]. This possibility is supported by data from animal models in which RAGE knock-out in animals fed a high-fat diet was sufficient to improve insulin tolerance in females but not in males [26]. However, it must not be overlooked the fact that RAGE can also recognize ligands other than AGEs. Thus despite the improvement in glucose homeostasis and insulin tolerance upon RAGE knockout in females but not in male mice, it cannot be inferred that this effect is strictly dependent on AGEs, considering RAGE can also bind other ligands [47]. However, these findings still suggest that females, relative to males, are more susceptible to the metabolically detrimental effects of RAGE ligands, including AGEs [26]. Another putative explanation for these sex-dependent effects is that the specific contribution of anthropometric parameters towards insulin resistance may differ between males and females. In keeping with this, not only the anthropometric variables able to predict HOMA-IR are different between males and females but in females the predictive power of these variables is lower compared to males and, most importantly, is increased by dietary AGEs. This suggests that insulin resistance in males, at least in this study cohort, is primarily dependent on anthropometric measures and particularly on fat mass and VAI. On the contrary in females, where the contribution of anthropometric variables is lower, dietary AGEs may further fuel insulin resistance promoted by the BMI and VAI.

From a mechanistic perspective, the relationship between AGE intake and insulin resistance does not appear to be related to NLRP3 inflammasome activation which, in turn, is pivotal for IL-18 production [48]. Indeed, despite this cytokine being associated with insulin resistance [49–51], its circulating levels are not related to dietary AGEs. However, this disagrees with the fact that AGEs have been reported to activate the NLRP3 inflammasome [52]. Nevertheless, there is also evidence that AGEs are able to attenuate NLRP3 inflammasome activation [53], a notion that reflects the lack of relationship between dietary AGEs and IL-18 circulating levels reported herein. Thus, the relationship between AGE intake and insulin resistance may be mediated by the activation of other pathways known to be triggered by AGEs [16–19, 42] even though this was not assessed as part of this study.

AGE intake correlated negatively with total and LDL-cholesterol only in females, whereas no relationship was found between these parameters in males. The negative relationship between dietary AGEs, total and LDL-cholesterol is surprising, particularly considering the tight relationship identified between the intake of AGEs and insulin resistance and the fact that a low intake of AGEs resulted in a decrease in total as well as LDL-cholesterol [54]. In light of this, the relationship between dietary AGEs and the circulating lipid profile in females warrants further studies.

This study has some limitations. In the first instance, circulating AGE levels were not assessed in order to confirm an overlapping between AGE intake and their plasma levels. Additionally, this study, to the same extent as other reports [22], did not discern between low and protein-bound AGE which are absorbed at different rates [55, 56]. Furthermore, this is an observational study which did not directly investigate whether modulating the intake of dietary AGEs via a nutritional intervention would differently impact upon insulin sensitivity in males and females. Always in line with this, the use of food frequency questionnaire may be prone to information bias [57], to reduce this bias, the questionnaire was administered by trained medical doctors with expertise in nutrition. Nevertheless, this study has also some strength in that it is the first study reporting a sex-dependent effect of dietary AGEs on insulin resistance. Additionally, the assessment of AGE intake was conducted using food frequency questionnaires as previously reported [22] which, in combination with published AGE databases [13, 28], have been proposed as the best method to estimate AGE intake in large cohorts [22]. An additional strength of the study is the assessment of habitual dietary habits in a large study cohort which allowed a reduction of information bias associated with food frequency questionnaires.

## Conclusion

To conclude dietary AGEs exert a sex-dependent effect on insulin resistance as their intake is associated and able to predict HOMA-IR in females but not males. This suggests that females may be more susceptible to the deleterious impact of these glycotoxins on insulin sensitivity. Nevertheless, considering this study not involving a nutritional intervention in order to directly elucidate whether the effect of AGEs on insulin resistance is sex-dependent, further studies are warranted in order to confirm the present findings.

## Supplementary Information

Below is the link to the electronic supplementary material.

Supplementary file1 (DOCX 32 KB)

## Data Availability

Data described in the manuscript, code book, and analytic code will be made available upon request pending. Data are not publicly available, due to the PANGeA study consortium agreement, which regulates the intellectual property of the data.

## Abbreviations

AGEs: Advanced glycation end-products
FFM: Fat-free mass
FM: Fat mass
hsCRP: High-sensitivity C-reactive protein
RAGE: Receptor for AGEs
VAI: Visceral adiposity index
