# Supplementary material for: Advanced glycation end-product intake predicts insulin resistance in a sex-dependent fashion
Source: Eur J Nutr. 2025 Apr 22;64(4):162. doi: 10.1007/s00394-025-03672-3 (PMC12014793; doi:10.1007/s00394-025-03672-3)
Supplement: Supplementary file 1 — Supplementary file1 (DOCX 32 KB) [file 394_2025_3672_MOESM1_ESM.docx]

**Table 1S.** General characteristics of the study population.

| **(n= 434)** | **Median (IQR)** |
| --- | --- |
| Age (years) | 66 (63 - 70) |
| BMI (kg/m^2^) | 26.25 (23.94 - 28.89) |
| Waist circumference (cm) | 92.00 (86.00 - 99.63) |
| FFM (%) | 64.70 (60.10 - 69.30) |
| FM (%) | 35.30 (30.70 - 39.90) |
| FFM Kg | 45.03 (38.63 - 54.14) |
| FM Kg | 24.68 (20.70 - 29.54) |
| Muscle Mass (Kg) | 27.90 (23.50 - 34.60) |
| VAI Index | 1.02 (0.70 - 1.50) |
| SBP (mmHg) | 136 (126 - 151) |
| DBP (mmHg) | 84 (77 - 92) |
| AGEs (kU/day) | 11523.1 (8419.2 - 15982.3) |
| Glucose (mg/dL) | 97 (90 - 106) |
| Insulin (U/L) | 7.9 (5.2 - 11.5) |
| HOMA-IR index | 1.9 (1.3 - 2.9) |
| Triglycerides (mg/dL) | 92 (71 - 118) |
| Total Cholesterol (mg/dL) | 215 (190 - 241) |
| Cholesterol HDL (mg/dl) | 65 (54 - 78) |
| Cholesterol LDL (mg/dl) | 129 (106 - 149) |
| hsPCR (mg/L) | 0.11 (0.06 - 0.23) |
| IL-18 (pg/ml) | 356.7 (281.6 - 448.2) |

BMI, body mass index; FFM, fat free mass; FM, fat mass; VAI, visceral adiposity index; SBP, systolic blood pressure; DBP, Diastolic blood pressure; AGEs, advanced glycation end products; HOMA-IR, homeostatic model assessment for insulin resistance; HDL, high density lipoprotein; LDL, low density lipoprotein; hsCRP, high-sensitivity C-reactive protein; IL-18, interleukin 18.

Table 2S. Energy and nutrient intake and diet quality of the study population.

|  | Male (n= 195) | Female (n= 239) |  |
| --- | --- | --- | --- |
|  | **Median (IQR)** | **Median (IQR)** | **p-value^1^** |
| Energy (kcal/day) | 1850.6 (1550.6-2103.5) | 1588.9 (1318.2-1882.0) | <0.001*** |
| Protein (g/day) | 71.6 (58.2-85.8) | 63.0 (50.0-77.4) | <0.001*** |
| Lipid (g/day) | 62.7 (49.0-76.9) | 58.3 (44.6-70.5) | 0.020* |
| Carbohydrate (g/day) | 219.1 (182.8-273.6) | 199.8 (154.1-245.1) | <0.001*** |
| Total fiber (g/day) | 18.7 (13.7-24.4) | 17.2 (13.9-22.3) | 0.143 |
| Cholesterol (mg/day) | 179.1 (120.5-256.6) | 156.1 (100.5-231.1) | <0.001*** |
| SFA (g/day) | 16.8 (11.7-23.0) | 15.6 (11.3-21.1) | 0.012* |
| PUFA (g/day) | 7.2 (4.9-10.0) | 6.1 (4.6-8.1) | 0.116 |
| MUFA (g/day) | 25.1 (19.5-32.5) | 24.0 (17.7-30.8) | 0.002** |
| MDA | 17.0 (15.0-18.0) | 17.0 (15.0-19.0) | 0.428 |

The comparison between males and females was carried out with Mann-Whitney test. *0.050>p–value≤0.010; **0.010>p–value<0.001; ***p–value≤ 0.001. SFA, Saturated fatty acids; PUFA, polyunsaturated fatty acids; MUFA, Monounsaturated fatty acids; MDA, Mediterranean Diet Adherence.

**Table 3S.** Pearson correlation between AGEs and anthropometric and metabolic parameters in the general population.

|  | **log AGEs (kU/day)** | |
| --- | --- | --- |
|  | **r** | **p-value** |
| log BMI (kg/m^2^) | -0.029 | 0.545 |
| log Waist circumference (cm) | 0.026 | 0.584 |
| log FM (Kg) | -0.071 | 0.142 |
| log FFM (Kg) | 0.135 | 0.005** |
| log VAI | -0.103 | 0.036* |
| log Triglycerides (mg/dL) | -0.109 | 0.027* |
| log Total Cholesterol (mg/dL) | -0.140 | 0.004** |
| log Cholesterol HDL (mg/dl) | -0.026 | 0.598 |
| log Cholesterol LDL (mg/dl) | -0.111 | 0.023* |
| log Glucose (mg/dL) | 0.041 | 0.406 |
| log Insulin (U/L) | 0.068 | 0.168 |
| log HOMA-IR index | 0.071 | 0.148 |
| log IL-18 (pg/ml) | 0.060 | 0.221 |
| log hsCRP (mg/L) | -0.027 | 0.588 |

*0.050>p–value≤0.010; **0.010>p–value<0.001. r, Pearson Correlation Coefficient; BMI, body mass index; FFM, fat free mass; FM, fat mass; VAI, visceral adiposity index; SBP, systolic blood pressure; DBP, Diastolic blood pressure; AGEs, advanced glycation end products; HOMA-IR, homeostatic model assessment for insulin resistance; HDL, high density lipoprotein; LDL, low density lipoprotein; hsCRP, high-sensitivity C-reactive protein; IL-18, interleukin-18.

**Table 4S.** Pearson correlation between HOMA-IR index and classical predictors for insulin resistance in the study population

|  | **log HOMA – IR index** | |
| --- | --- | --- |
|  | **r** | **p-value** |
| log BMI (kg/m^2^) | 0.495 | <0.001*** |
| log VAI | 0.368 | <0.001*** |
| log FM (Kg) | 0.490 | <0.001*** |

***p–value < 0.001. r, Pearson Correlation Coefficient; BMI, body mass index; FM, fat mass; VAI, visceral adiposity index.

**Table 5S.** Stepwise linear regression model indicating predictors of HOMA-IR index in study population.

| **Study population** | | | | | |
| --- | --- | --- | --- | --- | --- |
| **Model** | **Predictor** | **R^2^** | **Unstandardised B coefficient** | **Standard Error** | **p-value** |
| 1 |  | 0.243 | -2.896 | 0.326 | <0.001*** |
|  | log BMI |  | 2.244 | 0.230 | <0.001*** |
| 2 |  | 0.299 | -2.463 | 0.317 | <0.001*** |
|  | log BMI |  | 1.937 | 0.224 | <0.001*** |
|  | log VAI |  | 0.264 | 0.060 | <0.001*** |
| 3 |  | 0.311 | -3.058 | 0.346 | <0.001*** |
|  | log BMI |  | 1.935 | 0.192 | <0.001*** |
|  | log VAI |  | 0.277 | 0.045 | <0.001*** |
|  | log AGEs |  | 0.148 | 0.053 | 0.005** |
| 4 |  | 0.321 | -2.655 | 0.375 | <0.001*** |
|  | log BMI |  | 1.150 | 0.349 | 0.001*** |
|  | log VAI |  | 0.258 | 0.045 | <0.001*** |
|  | log AGEs |  | 0.159 | 0.052 | 0.003** |
|  | log FM |  | 0.479 | 0.178 | 0.007** |
| Dependent variable: log HOMA-IR | | | | | |
| Model 1 excluded variables: log AGEs (kU/day), log FM (Kg), log VAI.  Model 2 excluded variables: log AGEs (kU/day), log FM (Kg).  Model 3 excluded variables: log FM (Kg). | | | | | |

**0.010>p–value<0.001; ***p – value ≤0,001. BMI, body mass index; FM, fat mass; VAI, visceral adiposity index; AGEs, advanced glycation end products.
